# Around the Hospitals

**Published:** 1895-02-02

**Authors:** 


### Around the Hospitals.

[Notice to the Managers of Institutions.—Special space is now reserved for the insertion of notices of hospital meetings and festivals. The Editor requests that all notices may reach The Hospital Office, 428, Strand, at least one week before the date of each meeting.]

**West Sussex, East Hants, and Chichester General Infirmary and Dispensary.**—The infirmary has suffered, like many other institutions of the kind, from a decrease of funds under the heading of subscriptions and collections. The clergy have actively co-operated to assist as much as possible, and the committee have increased their efforts to keep down the expenditure, and by a careful comparison with the management of other infirmaries are able to determine where reduction can properly be looked for. We notice, too, that the secretary is not unmindful that in his department special economies are possible, and we observe that, no doubt with a view to lessening printing expenses, local trade advertisements find a place between matter and cover in the report. During the past year 336 in-patients and 1,294 out-patients were treated at the hospital. There is a Convalescent Fund in connection with the hospital, also another to provide flannel and trusses. The total receipts for the past year were £2,629, and the expenditure £2,970. The balance due to the treasurer at the end of the year was £1,033.

**The Kilmarnock Infirmary.**—We have to congratulate this institution on a thoroughly up-to-date and well drawn up report. Great improvements have been made since last year, which have rendered the document of increasing interest. The directors' report is printed in good clear type, the principal features and events of interest forming heads to the paragraphs dealing with them, a plan that should be universally adopted by secretaries who desire that their reports should be really read. Illustrations of the outside and the ground-plan of the hospital are given. Further, the directors show their desire to establish their institution in the van of progress, by adopting the uniform system of accounts, thus claiming the right to comparison with the best, large and small, hospitals throughout the United Kingdom. We regret that a report which bears the impress of excellent management should reveal that the ordinary income of the infirmary is not sufficient to meet the ordinary expenditure, and that there the clergy have not co-operated to the extent that they might have done in instituting congregational collections. We look forward with confidence to an improvement in this direction next year, feeling sure that the clergy of Kilmarnock will not fail to display the zeal so universal amongst their brethren elsewhere. The directors have not allowed scarcity of funds to interfere with necessary additions and reforms in the institution, and have consequently borrowed £1,000 to carry out necessary work. This debt we trust some generous friend will soon remove. The average number of patients treated has been exceptionally large during the year. The Convalescent Fund has been generously and anonymously augmented by a donation of £500. Several legacies have been received, and many gifts in kind show that interest in the infirmary is alive in the neighbourhood.

